# Lung epithelium development and airway regeneration

**DOI:** 10.3389/fcell.2022.1022457

**Published:** 2022-10-10

**Authors:** Evelien Eenjes, Dick Tibboel, Rene M.H. Wijnen, Robbert J. Rottier

**Affiliations:** ^1^ Department of Pediatric Surgery, Erasmus MC-Sophia Children’s Hospital, Rotterdam, Netherlands; ^2^ Department of Cell Biology, Erasmus MC, Rotterdam, Netherlands

**Keywords:** lung development, lung stem cells, regeneration, epithelial airway cells, airway biology

## Abstract

The lung is composed of a highly branched airway structure, which humidifies and warms the inhaled air before entering the alveolar compartment. In the alveoli, a thin layer of epithelium is in close proximity with the capillary endothelium, allowing for an efficient exchange of oxygen and carbon dioxide. During development proliferation and differentiation of progenitor cells generates the lung architecture, and in the adult lung a proper function of progenitor cells is needed to regenerate after injury. Malfunctioning of progenitors during development results in various congenital lung disorders, such as Congenital Diaphragmatic Hernia (CDH) and Congenital Pulmonary Adenomatoid Malformation (CPAM). In addition, many premature neonates experience continuous insults on the lung caused by artificial ventilation and supplemental oxygen, which requires a highly controlled mechanism of airway repair. Malfunctioning of airway progenitors during regeneration can result in reduction of respiratory function or (chronic) airway diseases. Pathways that are active during development are frequently re-activated upon damage. Understanding the basic mechanisms of lung development and the behavior of progenitor cell in the ontogeny and regeneration of the lung may help to better understand the underlying cause of lung diseases, especially those occurring in prenatal development or in the immediate postnatal period of life. This review provides an overview of lung development and the cell types involved in repair of lung damage with a focus on the airway.

## Introduction

After fertilization, tightly controlled processes and cell fate decisions guide the development of a full-grown organism from a single cell embryo. Axis formation and specification is followed by gastrulation, a highly complex process leading to the determination of the three germ layers, ectoderm, mesoderm and endoderm. The epithelial cells of the trachea, airway, and alveoli are derived from the endodermal lineage, whereas the lung mesoderm develops and generates various cell lineages like, vascular cells, smooth muscle cells, pericytes and cartilage precursors. The lung mesoderm and endoderm reciprocally interact, thereby affecting the development and differentiation of each other during all stages of development (Cardoso and Lu, 2006; Swarr and Morrisey, 2015). The pulmonary vasculature is already present early during lung development and expands as the lung grows (Canis Parera et al., 2005). Here, we will focus on the development of lung epithelium from endodermal progenitor cells.

## Origin and specification of the trachea and primary lung bud formation

**FIGURE 1:**
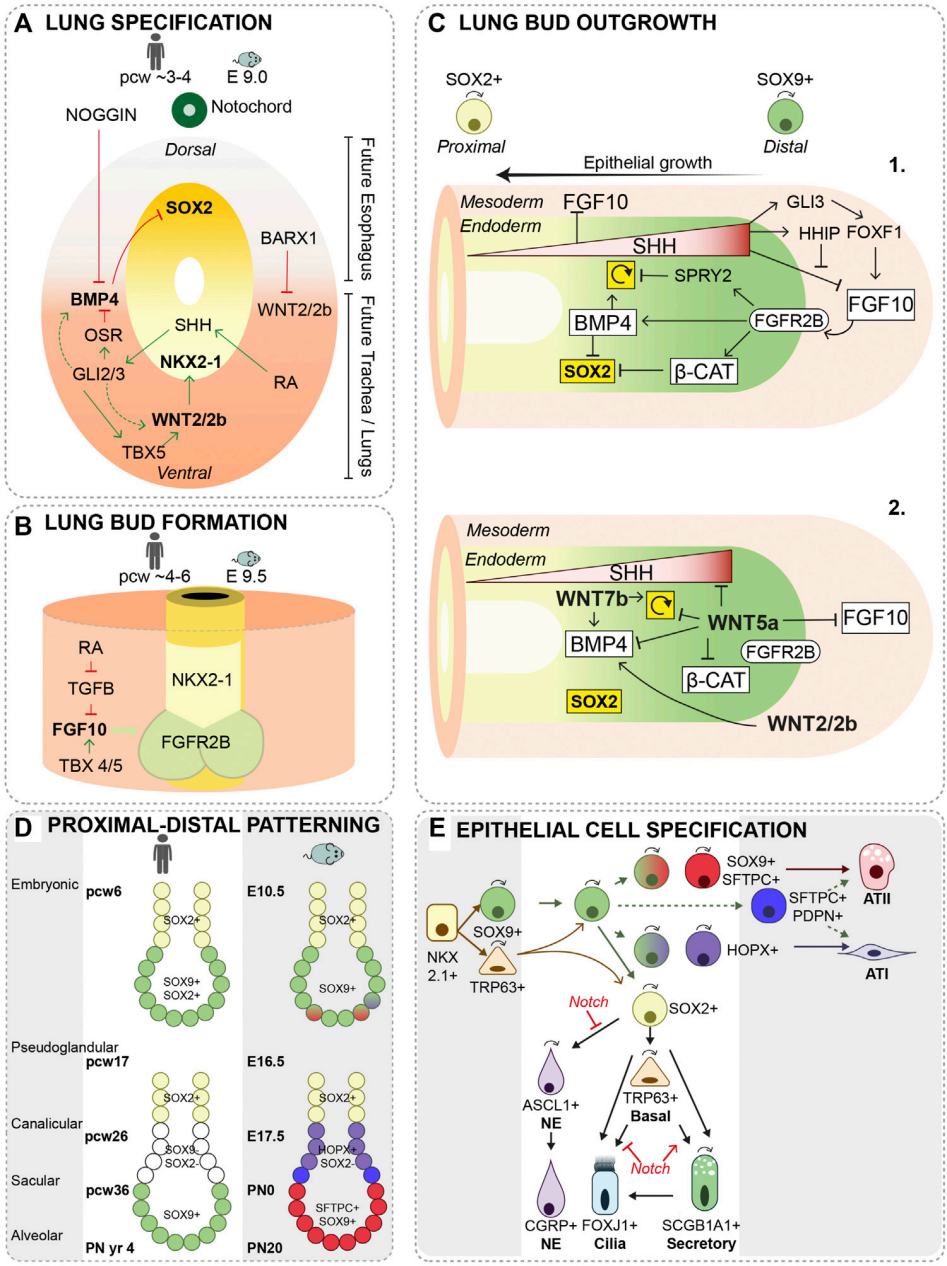
Lung specification, primary lung bud formation and growth. **(A)** During lung specification, *Nkx2*-*1* expression is restricted to the ventral side and *Sox2* to the dorsal side of the foregut endoderm. Retinoic acid (RA)-signaling activates RA receptors in the surrounding mesoderm driving cells to secrete Sonic Hedge Hog (SHH) in the ventral foregut mesoderm. SHH-responsive cells subsequently trigger activation of GLI2 and GLI3 transcription factors in the ventral mesoderm, which stimulate expression of WNT2/2b and BMP4 (Rankin et al., 2016). Odd-skipped related zinc finger transcriptional repressor, OSR, and SHH signaling target TBX5 are important modulators of WNT2/2b and BMP4 signaling (Han et al., 2017; Steimle et al., 2018). The transcription factor, BARX1, is expressed in the dorsal mesenchyme thereby repressing WNT signaling (Woo et al., 2011). **(B)** FGF10 from the ventral mesoderm is essential in lung bud formation, and is regulated by RA and TGF-β signaling. TBX transcription factors present in the foregut mesoderm has shown to be essential in regulating FGF10 expression as well (Sakiyama et al., 2003; Arora et al., 2012). E = embryonic day, pcw = post-conceptional week **(C-1)** Several reciprocal interactions between mesoderm and endoderm regulate the expansion of the distal tip through proliferation and suppression of *Sox2* expression. SHH is expressed in a gradient with the highest expression in the distal bud. SHH inhibits mesenchymal FGF10 expression just proximal of the distal bud. At high concentrations, SHH induces expression of, HH inhibitory protein (HHIP) in the distal mesenchyme to allow for FGF10 expression *via* regulation of GLI3 and FOXF1 (Morrisey and Hogan, 2010). Proliferation of progenitor cells is positively regulated *via* BMP4 induction or inhibited *via* its antagonist SPRY2 (Weaver et al., 2000; Mailleux et al., 2001; Hyatt et al., 2004; Eblaghie et al., 2006). *Sox2* expression is inhibited *via* Wnt-β-Catenin and BMP4 signaling (Volckaert et al., 2013; Wang et al., 2013). ↻ = proliferation. **(C-2)** Knock-out mouse models of WNT ligands demonstrated defects in lung development; WNT2/2b (canonical) (Hrycaj et al., 2015) in distal mesenchyme, WNT5a (non-canonical) (Li et al., 2002; Li et al., 2005; Volckaert and De Langhe, 2015) and WNT7b (canonical) in distal epithelium (Rajagopal et al., 2008), each suggested to be involved in the regulation of BMP4, β-Catenin, SHH signaling or cell proliferation. ↻ = proliferation. **(D)** A proximal-distal patterning of the lung bud regionalizes the airway epithelium during branching. In human, distal bud progenitor cells are characterized by *SOX9* and *SOX2* expression, while in mice these cells only express *Sox9.* After the pseudoglandular stage, a more similar pattern is present with, SOX9*+* progenitors present in the tip of the distal bud, SOX2- SOX9-just proximal of the distal bud and SOX2+ progenitors in the proximal airways. In mice, the patterning of the distal bud is further specified by the expression of *Sftpc* and *Hopx.* **(E)** During growing of the primary lung buds and the pseudoglandular stage, SOX9+ progenitor cells give rise to the SOX2+ airway progenitor and a few basal cells are present. Some SOX9+ progenitors start to specify to alveolar type (AT) I or ATII cells around E13.5. SOX2+ progenitors differentiate to neuroendocrine cells (NE) and basal cells. The basal cells that develop at this stage, can self-renew and differentiate to ciliated and secretory cells in the extrapulmonary airways. SOX2+ progenitor cells also further differentiate to secretory and ciliated cells. Secretory cells can self-renew and give rise to ciliated cells (Rawlins et al., 2009b). Notch signaling inhibits or stimulates different cellular specifications at different stages of lung development. During the canalicular and saccular stage, the terminal buds become narrower and numerous alveolar sacs develop that are the precursors of the alveoli. SFTPC+SOX9+, SFTPC+HOPX+ and HOPX^+^ progenitor cells further differentiate into ATI or ATII cells. At the end of embryonic lung development, which continues postnatally, the alveolar sacs are subdivided by the formation of secondary septae and the ATI cells become closely associated with the endothelial cells, forming a thin layer allowing for gas exchange (Morrisey and Hogan, 2010). ↷ cell division, E = embryonic day, pcw = post-conceptional week, PN = post-natal.

Specification of lung and esophagus starts from the anterior foregut endoderm. Sry-related HMG box 2 positive (SOX2+) dorsal esophagus progenitors are separated from ventral, NK2 Homeobox 1 positive (NKX2-1+) lung progenitors (Minoo et al., 1999; Que et al., 2007) (for details see Figure 1A). Reciprocal signaling cues between mesoderm and endoderm contribute to a proper localization of *Nkx2*-1 expression (Figure 1A) (Swarr and Morrisey, 2015; Billmyre et al., 2015; Morrisey and Rustgi, 2018; Kishimoto et al., 2018; Kiyokawa and Morimoto, 2021). *Nkx2-1* expression is induced by canonical Wingless and Int1 2 (WNT2) and WNT2b ligands from the ventral mesoderm and by Fibroblast Growth Factor 2 (FGF2) secretion from adjacent developing cardiac mesoderm (Serls et al., 2005; Goss et al., 2009; Harris-Johnson et al., 2009). *Sox2* expression is repressed in the ventral foregut endoderm due to the secretion of Bone Morphogenetic Protein 4 (BMP4) from the ventral mesoderm (Domyan et al., 2011). The BMP antagonist NOGGIN is secreted by cells of the notochord, suppressing BMP signaling in the dorsal mesoderm and allowing *Sox*2 expression (Que et al., 2006; Li et al., 2007). SOX2 represses *Nkx2-1* expression, thereby restricting its expression to the ventral foregut endoderm (Figure 1A) (Domyan et al., 2011). In addition, canonical WNT signaling induces *Wnt7b* expression in the endoderm, which in turn activates *Tbx4* in the surrounding mesoderm (Kishimoto et al., 2020). T Box transcription factor 4 (TBX4) activates the branch inducing growth factor, FGF10, and is involved in fibroblast maturation (Sakiyama et al., 2003; Masafumi et al., 2018). Inactivation of mesodermal WNT signaling leads to cartilage agenesis as well as malformation of the circumferential smooth muscle cell layer (Kishimoto et al., 2020).

SOX2 and NKX2-1 demarcate the Dorsal-Ventral (D-V) boundary of the foregut endoderm and are important in separating the trachea from the esophagus. Mouse models with reduced expression *Sox2* or absence of *Nkx2*-1 resulted in separation defects, resembling the human congenital condition called tracheoesophageal fistula (TEF), where the airway is connected with the stomach and/or esophageal atresia (EA), a short and blunted esophagus (Minoo et al., 1999; Que et al., 2006; Que et al., 2007). Multiple factors contributing to trachea and esophagus D-V patterning have been identified using genetic mouse models, such as Nkx2-1^−/−^ (Canis Parera et al., 2005), Sox2^GFP/COND^ (Que et al., 2007), Bmp4^COND^ (Li et al., 2008), Barx1^−/−^ (Woo et al., 2011), Noggin^−/−^ (Que et al., 2006; Li et al., 2007), Gli2/3 (Motoyama et al., 1998) and Shh (Litingtung et al., 1998; Pepicelli et al., 1998), or through genetic screens of human infants born with EA/TEF, such as NOGGIN (Murphy et al., 2012) and SOX2 (Williamson et al., 2006) (Figure 1A) (Que et al., 2006; Billmyre et al., 2015). Although genetic analyses of human EA/TEF patients and animal models revealed genes associated with EA/TEF, the cellular mechanisms causing the separation defect are poorly understood (Brosens et al., 2020; Brosens et al., 2021).

After specification of lung progenitors, the single common foregut tube begins to compartmentalize (Cardoso and Lu, 2006; Schittny, 2017; Whitsett et al., 2019; Zepp and Morrisey, 2019). A timed and localized expression of retinoic acid (RA) induces mesenchymal expression of FGF10, which activates NKX2-1+ lung progenitor cells by binding to its receptor FGFR2B and subsequently induces lung bud formation (Malpel et al., 2000; Desai et al., 2004; Chen et al., 2007) (Figure 1B). At the same time of lung bud formation, the trachea, separates from the esophagus proximal of the lung buds (Que et al., 2006; Kishimoto et al., 2018; Nasr et al., 2019). Of note, FGF10 knock out mice show normal formation of the trachea, while the lung buds do not form (Bellusci et al., 1997; Sekine et al., 1999), suggesting that a distinct mechanism of FGF10 signaling is involved in formation and separation of the trachea from the esophagus. Recently, single cell RNA sequencing of the mouse foregut expands the identification of cell types and the identification of reciprocal interactions between endoderm and mesoderm, as well as lineage relationships between cell types (Han et al., 2020).

## Branching morphogenesis

A complex tree-like structure of airways is formed at the pseudoglandular stage, with a repetitive pattern of formation of new buds, bifurcation and outgrowth of buds (Metzger et al., 2008). During branching of the airways, SOX9+ Inhibitors of DNA binding 2+ (ID2+) progenitor cells reside at the branching distal tips. These tip progenitors, are multipotent and give rise to the SOX2+ progenitor cells which will form the airway epithelium (Gontan et al., 2008; Rawlins et al., 2009a; Que et al., 2009). In contrast to the mouse branching airways, in human lung the tip progenitors express both SOX9 and SOX2 (Figure 1D) (Nikolic et al., 2017; Danopoulos et al., 2018; Eenjes et al., 2021).

Maintaining a proximal-distal patterning during lung development is crucial for a proper branching of the airways. We previously illustrated formation of cystic airway structures in a mouse model where *Sox2* expression was induced in the distal tip progenitor cells (Gontan et al., 2008). During the last decades, the use of transgenic mouse models contributed highly to the identification of multiple epithelial-mesenchymal signaling pathways important for maintaining a proximal-distal patterning and coordinating initiation and outgrowth of lung buds [see (Morrisey and Hogan, 2010; Whitsett et al., 2019; Zepp and Morrisey, 2019) and Figure 1C]. FGF10 is important for primary bud formation, and continues to be present in the mesenchyme surrounding the outgrowing buds during branching morphogenesis (Bellusci et al., 1997; Yuan et al., 2018). The localized source of FGF10 within the “tip-microenvironment” regulates multiple factors to control expansion of the bud by inducing proliferation and suppressing *Sox2* expression to prevent differentiation (Figure 1C) (Hyatt et al., 2004; Shu et al., 2005; Volckaert et al., 2013; Wang et al., 2013; Chao et al., 2019). As the lung bud grows, cells become displaced from the FGF10 source and differentiate to SOX2+ airway progenitor cells. FGF10 plays a central role in branching morphogenesis of mouse lungs, however, FGF10 is not essential for branching of human fetal lungs *in vitro* (Danopoulos et al., 2019).

## Development of proximal airway and distal alveolar lineages

During branching morphogenesis, SOX2+ progenitor cells proliferate but also start to differentiate into proximal airway cell lineages (Figure 1D). SOX2 positive cells demarcates the airway epithelium after progenitor cells differentiate, and deletion of SOX2 during development shows a severe reduction in basal, ciliated and secretory cells (Que et al., 2009).

Differentiation commences with the appearance of a few basal cells (Transformation-related protein 63) at E9.5 in the trachea and in proximal regions of the lung bud in mice. Lineage tracing studies using *Trp63-CreERT* shows that presumptive basal cells genetically labeled before E9.5 give rise to both airway and alveolar epithelial cells (Figure 1D). Lineage-labeling from E10.5 onward showed that the basal cells only serve as progenitors for the cells in the pseudostratified epithelium of the extrapulmonary airways (trachea and main bronchi) (Figure 1D) (Yang et al., 2018). *Vice versa*, lineage tracing of tip progenitor cells using *Sox9-Cre* or *Id2-Cre* induced before E9.5, shows that tip progenitor cells give rise to airway epithelial cells both in the extra- and intra-pulmonary airways, whereas induction at E11.5 shows that tip progenitor cells only give rise to the intrapulmonary airways (Rawlins et al., 2009a; Yang et al., 2018). So, during lung specification and lung bud formation (E8.5–E9.5), two complementary lineages are defined early in trachea/lung development, both contributing to the epithelial cells of the respiratory tract.

At E13.5, as the bronchial tree is expanding, SOX2+ progenitor cells give rise to neuroendocrine (NE) cells and non-NE cells (Figure 1E). Precursors of NE cells, are first scattered throughout the proximal airway epithelium and subsequently migrate to form NE clusters, which are mostly located at the bifurcations of airways (Kuo and Krasnow, 2015; Noguchi et al., 2015; Noguchi et al., 2020). Notch activity controls the choice between NE and non-NE cell fate (Ito et al., 2000; Jia et al., 2015; Shue et al., 2022). Inhibition of Notch signaling results in an increase in NE cells, but also in an increase in ciliated cells at the expense of secretory cells. This showed that at later stages in development (after E15.5), Notch signaling balances the differentiation between secretory and ciliated cells (Figure 1E) (Rawlins et al., 2007; Tsao et al., 2009; Morimoto et al., 2012). NE cell hyperplasia is associated with CDH, but whether this contributes to the onset or specific pathology related to CDH is not yet investigated (Ijsselstijn et al., 1997). Previously, it was shown that overexpression of *Sox2* during lung development resulted in increased basal cell numbers, but also to an increase in NE cells. However, the underlying molecular mechanisms that guide the SOX2+ airway progenitor to differentiate to basal or NE cells is not yet understood (Gontan et al., 2008).

**FIGURE 2:**
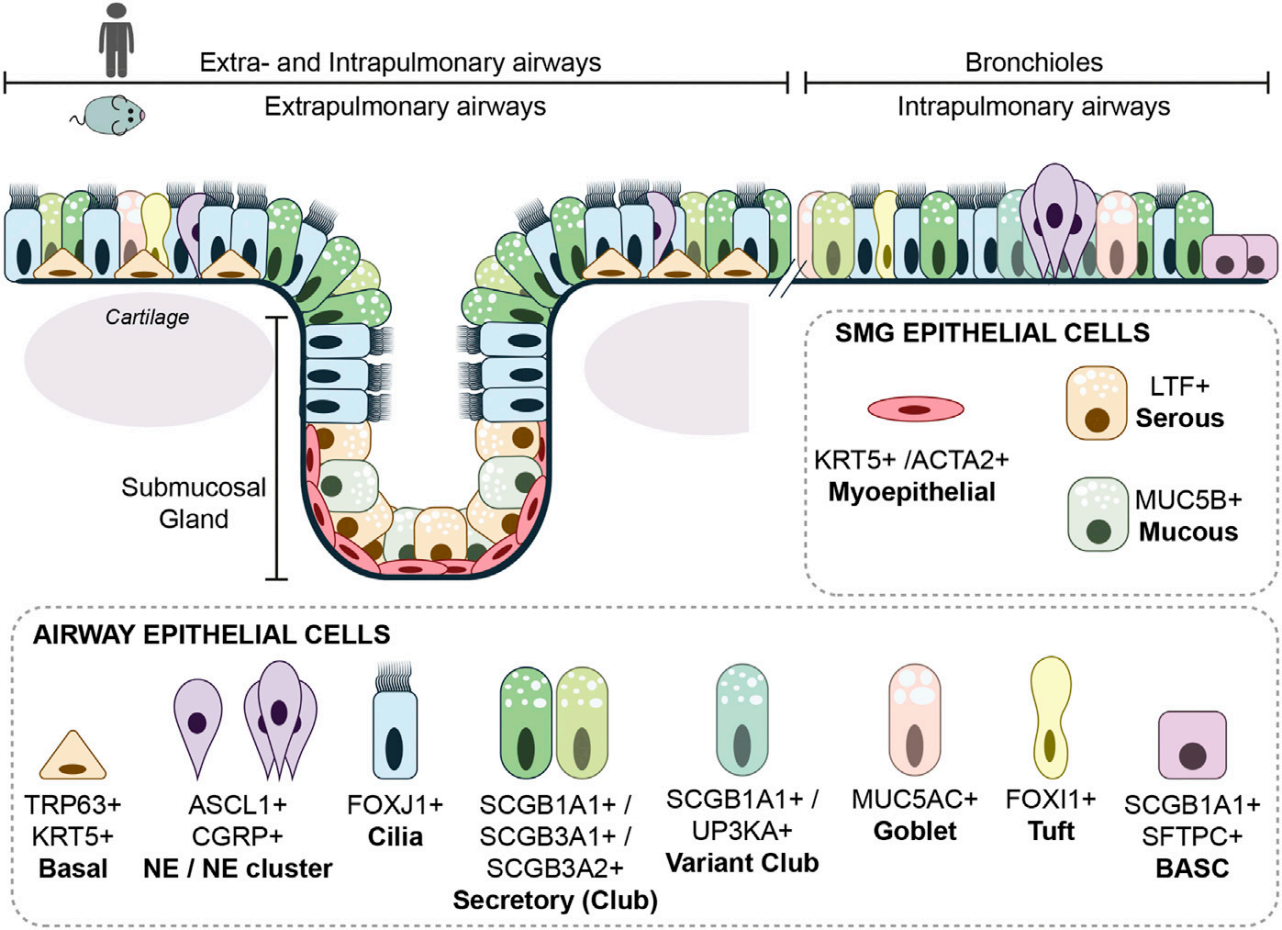
Cellular composition of the airways. The extrapulmonary epithelium (trachea and main bronchi) of the mouse consists of a pseudostratified epithelium containing basal cells, while in the human lung, basal cells are only absent in the bronchioles proximal of the alveoli. Submucosal glands are in mouse and human only present in cartilaginous airways. The airway epithelium consists of many different epithelial cells, the main epithelial cells are described within the text. A rare epithelial cell type is the ionocyte. Ionocytes appear to be the main source of cystic fibrosis transmembrane conductance regulator (CFTR) activity, thereby regulating mucous production (Montoro et al., 2018). Goblet cells are nearly absent in mouse airway epithelium, but are more frequently found in human airway epithelium and are together with other secretory cells, responsible for mucous production.

Mature alveoli exist of cuboidal surfactant producing alveolar type 2 cells (ATII) and flattened alveolar type I (ATI) cells. The first specification of SOX9+ tip progenitors to either ATI or ATII cells is observed at E13.5 (Figures 1D,E) (Frank et al., 2019). From E15.5 onward, SOX9+ progenitors are still involved in branching of distal tips, but cells in this recently branched epithelium do not express SOX2, as they do early in development, but rather express the ATI marker, Homeodomain-Only Protein homeobox (HOPX) (Figure 1D) (Alanis et al., 2014; Frank et al., 2019). In addition, bipotent progenitor cells expressing both ATI and ATII markers, can be found in the distal bud but they show only minor contribution to the alveolar compartment during development (Figure 2) (Desai et al., 2014; Treutlein et al., 2014; Frank et al., 2019; Zepp et al., 2021). In human lung development, distal tip progenitors loose *SOX2* expression and remain only SOX9+ in the canalicular and saccular stage (Figure 1D). However, tip progenitor cells already start to express both markers of ATI and ATII cells 5 weeks prior to the canalicular stage and in co-expression with SOX2 (Nikolic et al., 2017). The functional significance of *SOX2* expression in human tip progenitor cells during the pseudoglandular stage is currently unknown.

## Epithelial lineage diversification and cell plasticity upon airway regeneration

As a result of lung development, the airway epithelium is aligned with a wide range of cell types (Figure 2). During steady state, the airway epithelium is a low turnover tissue, but upon severe damage, quiescent progenitor cells can regenerate the airway epithelium. Lineage tracing studies in mice demonstrated that within the airway epithelium, most adult epithelial cells retain plasticity to dedifferentiate or transdifferentiate under stress or damage conditions. Ciliated cells seems to be an exception, which have no apparent potential to proliferate or differentiate after injury (Rawlins et al., 2007) The interaction with the underlying mesenchyme and vasculature is important in the differentiation and regeneration of the epithelium [reviewed in (Mammoto and Mammoto, 2019; Tsuchiya et al., 2020)]. For instance, Dll4 deficient mice resulted in microvascular defects and subsequent impaired alveolarization (Xia et al., 2021). In mouse model of regenerative alveolarization, it was shown that capillary endothelial cells were stimulated to secrete growth factors that would induce epithelial proliferation (Ding et al., 2011). Recent work has described distinct processes and specialized AT2 cells that contribute to alveolar regeneration after induced damage in mice (Paisley et al., 2014; Choi et al., 2020; Kobayashi et al., 2020; Hurskainen et al., 2021). Like for the alveolar compartment, the epithelial cells of the airways are also subjected to signaling from the underlying mesenchymal cells. Upon injury, epithelial cells secreted Wnt7b, which subsequently induced the mesenchymal smooth muscle cells to express Fgf10 and thereby activating the basal cells (Volckaert et al., 2017). Here, we focus on the main adult airway cell types that are known to contribute to repair after injury. A more extensive description of lung regeneration and *in vitro* models to study adult airway epithelium was reviewed previously (Schilders et al., 2016; McQualter, 2019).

## Basal cells

The basal cell is one of the most studied cell types of the lung regarding regeneration. In mouse lung, basal cells are mainly located in the extrapulmonary airway epithelium, while the distribution in the human lung ranges from the trachea down to the smallest airways (Figure 2) (Rock et al., 2010). *In vitro* cultures using isolated mouse and human basal cells has shown that these cells could self-renew and are multipotent, meaning that they could differentiate to secretory and ciliated cells (Rock et al., 2009; Eenjes et al., 2018).

Human and mouse basal cells are characterized by the expression of *Trp63*, and *Trp63* knock-out mice completely lack basal cells (Mills et al., 1999; Yang et al., 1999; Daniely et al., 2004). Besides *Trp63* expression, all basal cells also express *Cytokeratin 5* (*Krt5*), and a subpopulation of basal cells express *Cytokeratin 14* (*Krt14*), which greatly expands upon injury (Hong et al., 2004a; Hong et al., 2004b). In human airway epithelium, *KRT14* also shows a more restricted expression pattern than *KRT5*, but increases in regions of squamous metaplasia in COPD patients (Rock et al., 2010). However, a functional difference between KRT14+ and KRT14-basal cells is not yet explored. Furthermore, basal cells are thought to be the source of lung squamous cell carcinoma through increased expression of both *SOX2* and *TRP63* (Bass et al., 2009; Ferone et al., 2016). The regulation of basal cell maintenance, proliferation and differentiation in relation of SOX2 is poorly understood, although ectopic expression of SOX2 induced the emergence of basal cells (Gontan et al., 2008; Kapere Ochieng et al., 2014; Ochieng et al., 2014). Recent single cell RNA sequencing data revealed that potentially several basal cells, or basal-like cells exist in the lung, that could form a continuum of differentiation (Montoro et al., 2018; Plasschaert et al., 2018; Travaglini et al., 2020; Basil et al., 2022; Kadur Lakshminarasimha Murthy et al., 2022).

A very small population of *Trp63* expressing cells reside in the mouse intrapulmonary airways. The number of these distal basal cells substantially increases upon severe lung injury. Lineage tracing showed that these cells contributed to both alveolar and airway lineages, showing the high potential of distal TRP63+ cell population (Vaughan et al., 2015; Zuo et al., 2015; Yang et al., 2018). Although, a similar population of basal cells was identified in human terminal bronchioles, its expansion or differentiation potential and contribution to airway regeneration is still uncertain (Vaughan et al., 2015).

## Submucosal glands

Submucosal glands (SMGs) are specialized secretory glands with a grape like structure embedded within the connective tissue, just underneath the proximal tracheal epithelium of the mouse and the cartilaginous airways of the human (Figure 2) (Tata and Rajagopal, 2017). The submucosal glands can be subdivided in the ducts and acini. The ducts contain a similar cellular composition as the surface epithelium of the airways. The acini contain basally located myoepithelial cells expressing *Krt14*, *Krt5*, and smooth muscle actin 2 (*Acta2*), and luminal cells secreting mucous and fluids rich in antimicrobial enzymes (Hegab et al., 2011; Lynch and Engelhardt, 2014). Upon injury, basal myoepithelial cells migrate to the surface epithelium of the trachea and aid in repopulating the airway due to proliferation and differentiation to basal, ciliated and secretory cells (Lynch et al., 2018; Tata et al., 2018). In pigs, similar to human, SMGs are present throughout the cartilaginous airways and exposure to chlorine gas showed that SMG derived cells contributed to the repair of the airway (Tata et al., 2018).

## Secretory cells

Secretory (Club) cells produce mucins and microbial peptides to capture inhaled substances, which are propelled out of the lung through cilia movement. Different subsets of secretory cells in mouse and human airways are identified by the secretion of different members of secretoglobins; SCGB1A1, SCGB3A1 or SCGB3A2 (Reynolds et al., 2002) (Figure 2). Lineage tracing studies, using secretory cell marker SCGB1A1, showed that besides the protective function, secretory cells have the potency to self-renew, differentiate to ciliated cells, and de-differentiate to basal cells (Rawlins et al., 2009b; Tata et al., 2013).

Naphthalene-induced injury is a frequently used mouse model to study airway regeneration (Van Winkle et al., 1995). Secretory cells are most vulnerable to naphthalene exposure due to their expression of cytrochrome P450 enzyme (*Cyp2f2*), which converts naphthalene to a cytotoxic product (Plopper et al., 1992). A subset of secretory cells, the variant club cells, was identified because they lack *Cyp2f2* expression, and survive naphthalene exposure (Reynolds et al., 2000; Hong et al., 2001). The variant club cell is closely located to neuroendocrine cell clusters, and expresses besides *Scgb1a1*, also *Uroplakin3a* (*UPK3a*) (Figure 2) (Guha et al., 2017). A similar localization of UPK3a+ secretory cells near neuroendocrine cells was observed in human lung sections, suggesting a similar progenitor cell population might be present (Guha et al., 2017).

## Neuroendocrine cells

Neuroendocrine (NE) cells are a rare population of cells in the airway epithelium and act as chemosensory cell, communicating with the nervous system and influencing smooth muscle tone as well as regulating immune response (Branchfield et al., 2016; Sui et al., 2018; Garg et al., 2019; Noguchi et al., 2020). NE cells also have the ability to contribute to airway epithelial repair after naphthalene induced injury (Song et al., 2012; Ouadah et al., 2019). As mentioned, hyperplasia of NE cells has been implicated in a number of lung diseases, which some of them are pediatric lung diseases, like BPD and CDH (Ijsselstijn et al., 1997; Cutz et al., 2007). Furthermore, NE cell markers are found in small cell lung cancer (SCLC) (van Meerbeeck et al., 2011), and *in vivo* studies in mouse showed the NE cells are the origin for SCLC development (Song et al., 2012; Ouadah et al., 2019). How and why NE cells associate with such a wide range of lung diseases is unknown and therefore an interesting airway population to study.

## Bronchioalveolar stem cells

In the zone where bronchiole transition to the alveoli, epithelial cells reside carrying both the secretory cell marker SCGB1A1 and ATII marker SFTPC (Kim et al., 2005) (Figure 2). These, so called Broncho-Alveolar Stem Cells (BASCs), showed self-renewal potential and were able to differentiate to bronchiolar and alveolar cell types *in vitro* (Kim et al., 2005; Lee et al., 2014; Lee et al., 2017). A recent dual-lineage tracing approach, showed that SFTPC+ SCGB1A1+ cells contribute to bronchiolar and alveolar epithelium after naphthalene-induced airway injury or bleomycin-induced alveolar injury, respectively (Liu et al., 2019; Salwig et al., 2019). However, BASCs are relatively stable in normal lung homeostasis, showing that BASCs are only activated upon injury (Liu et al., 2019; Salwig et al., 2019). In addition, lineage tracing studies using *Scgb1a1-Cre* showed that; SCGB1A1+ cells did not contribute to alveolar repair after hyperoxic aveolar injury (Rawlins et al., 2009b), suggesting that contribution of SCGB1A1+ cells to alveolar repair depends on the type and possibly severity of injury. Interestingly, recently a progenitor cell was described residing in the human terminal and respiratory bronchioles that shared an expression profile with SCGB1A1+ secretory cells and AT2 cells (Basil et al., 2022; Kadur Lakshminarasimha Murthy et al., 2022).

In conclusion, airway epithelial cells have a great ability to regenerate the airway epithelium and the contribution of different cell types can be assessed by the use of lineage tracing tools, and different injury models. However, the identification of progenitor lineages is much faster than the understanding of underlying mechanisms in the contribution of each cell type to regeneration. Increasingly sensitive methods, such as single cell RNA sequencing, spatial transcriptomics, ATAC-sequencing, and other multi-omics approaches, are being employed to analyze improved and newer models to study the role of the different cell types in development and regeneration (Krassowski et al., 2020; Subramanian et al., 2020). Furthermore, most of airway epithelial cell plasticity is observed in mouse models, translating these findings to either; the quiescent human airway epithelium; or the mis-regulation of cellular plasticity upon disease will be a great challenge. Importantly, the fast growth in the development of *in vitro* lung models, such as lung organoids, air-liquid interphase cultures and lung-on-a-chip model, may contribute to increase our understanding of human airway plasticity in development, homeostasis and disease (Schilders et al., 2016; McQualter, 2019).
